# Prevalence of Enteropathogens and Virulence Traits in Brazilian Children With and Without Diarrhea

**DOI:** 10.3389/fcimb.2020.549919

**Published:** 2020-09-25

**Authors:** Victor R. Merino, Viviane Nakano, Sabine Delannoy, Patrick Fach, Gabriela G. F. Alberca, Mauricio J. Farfan, Roxane M. F. Piazza, Mario J. Avila-Campos

**Affiliations:** ^1^Laboratório de Anaeróbios, Departamento de Microbiologia, Instituto de Ciências Biomédicas, Universidade of São Paulo, São Paulo, Brazil; ^2^Agence Nationale de Sécurité Sanitaire de l'Alimentation, de l'Environnement et du Travail, Food Safety Laboratory, Université Paris-Est, Champs-sur-Marne, France; ^3^Departamento de Pediatría y Cirugía Infantil, Facultad de Medicina, Campus Oriente-Hospital Dr. Luis Calvo Mackenna, Universidad de Chile, Santiago, Chile; ^4^Laboratório de Bacteriologia, Instituto Butantan, São Paulo, Brazil

**Keywords:** enteric pathogens, *Escherichia coli* pathotypes, diarrheal children, healthy children, molecular diagnostics

## Abstract

The use of molecular diagnostics for pathogen detection in epidemiological studies have allowed us to get a wider view of the pathogens associated with diarrhea, but the presence of enteropathogens in asymptomatic individuals has raised several challenges in understanding the etiology of diarrhea, and the use of these platforms in clinical diagnosis as well. To characterize the presence of the most relevant bacterial enteropathogens in diarrheal episodes, we evaluated here the prevalence of diarrheagenic *E. coli* pathotypes, *Salmonella* spp., and *Yersinia enterocolitica* in stool samples of children with and without diarrhea using real-time quantitative PCR (qPCR). We found that the presence of genetic markers associated with bacterial pathogens was significantly higher in stool samples from the diarrhea group compared to the control (*P* < 0.001). Bacterial loads in samples positive for *eae* and *aggR* markers were also determined. Compared to samples from asymptomatic children, a significantly higher number of copies of the *eae* gene were found in diarrhea samples. Also, the presence of genetic markers associated with STEC strains with clinical significance was evaluated in *eae*-positive samples by high-throughput real-time PCR. The data presented herein demonstrated that asymptomatic children of an urban area in Brazil might be enteropathogen reservoirs, especially for STEC.

## Introduction

Diarrheal disease is the second most important cause of child morbidity and mortality in the world, and is frequently due to contaminated food and water. Worldwide, 780 million individuals lack access to safe drinking water and 2.5 billion lack adequate sanitation. Diarrhea is widespread throughout developing countries. In low-income countries, children under 3 years old experience on average three episodes of diarrhea every year (World Health Organization, 2017).

Rotavirus and diarrheagenic *Escherichia coli* (DEC) are the two most common etiological agents of moderate-to-severe diarrhea in low-income countries (World Health Organization, 2017). Other pathogens such as Cryptosporidium, Shigella, *Campylobacter, Salmonella*, and *Yersinia* species also cause human bacterial gastroenteritis, but location-specific etiological patterns also need to be considered (Bottone, 1999; Freitas Neto et al., 2010; Marinou et al., 2012; Nunes et al., 2012; World Health Organization, 2017; Lima F. M. et al., 2019; Leli et al., 2020).

Enterotoxigenic *E. coli* (ETEC), enteroinvasive *E. coli* (EIEC), typical and atypical enteropathogenic *E. coli* (tEPEC and aEPEC), typical and atypical enteroaggregative *E. coli* (tEAEC and aEAEC), and Shiga toxin-producing *E. coli* (STEC) and its subgroup enterohemorrhagic *E. coli* (EHEC), enteroinvasive *E. coli* (EIEC), and diffusely adherent *E. coli* (DAEC) comprise DEC pathotypes (Kaper et al., 2004; Gomes et al., 2016). These pathogens use many common features to colonize the intestinal mucosa and trigger disease, but its initiation, progression, and complications vary considerably (Croxen et al., 2013). Thus, ETEC isolates carry the enterotoxins LT and/or ST on plasmids, as well as colonization factors (CFs). tEPEC, aEPEC, and LEE-positive STEC strains (EHEC) carry the locus of enterocyte effacement (LEE) which contains *eae*, the gene that encodes intimin, but only tEPEC has the bundle-forming pilus gene (bfp). STEC strains, LEE-positive and -negative, carry the Shiga toxin genes (stx1 and/or stx2). Some virulence factors for EAEC for a certain group of isolates are found on the pAA plasmid, and the presence of the *aggR* gene differentiates tEAEC from aEAEC. EIEC and also *Shigella spp*. have the ability to invade cells mainly through the pINV plasmid and acquired additional virulence attributes mainly from chromosomal pathogenicity islands (PAIs) (Croxen et al., 2013).

Nevertheless, the designation of certain isolates into a pathotype is complicated due to the plasticity of the *E. coli* genome resulting in the emergence of hybrid strains; besides, DEC epidemiology varies in different regions of the world and may depend on the host and environmental factors (Croxen et al., 2013). It is important to note that DEC pathotypes have been largely disregarded because of the lack of laboratory capabilities (Piazza et al., 2010; Miliwebsky et al., 2016). Moreover, in several case-control studies, it shows almost equal distribution, especially in EPEC and EAEC pathotypes (Bueris et al., 2007; Hernandes et al., 2009; Gomes et al., 2016; Imdad et al., 2018; Dias et al., 2020).

Besides LEE-positive pathogens and *Shigella, Salmonella* also uses the type III secretion system (T3SS) apparatus to deliver effector proteins into the host cell, and *invA*, an inner-membrane component, is critical to the functioning of *Salmonella* T3SS (Galán et al., 1992; Ginocchio and Galán, 1995). In addition, the most common virulence-associated gene in pathogenic Yersinia enterocolitica proved to be *ystA*, which can therefore be considered the best target gene to be amplified to evaluate the presence of pathogenic biotypes (Peruzy et al., 2017).

The use of multiple pathogen detection platforms in epidemiological studies have allowed us to get a broader view of the pathogens associated with diarrhea, but the presence of enteropathogens in asymptomatic individuals has raised several challenges in understanding the etiology of diarrhea, as well as the use of these platforms in clinical diagnosis (Liu et al., 2012; Lima A. A. M et al., 2019). The use of real-time quantitative PCR (qPCR) to determine bacterial loads in stool samples appears as an alternative to associate a pathogen with diarrheal cases.

To determine the most prevalent bacterial pathogens of diarrhea in children, we used qPCR to detect and quantify the presence of EPEC, ETEC, EAEC, STEC, EIEC, *Shigella, Salmonella*, and *Y. enterocolitica*. Furthermore, in a subset of *eae*-positive samples, we analyzed by high-throughput real-time PCR the presence of genetic markers related to EHEC strains with clinical significance.

## Materials and Methods

### Sample Collection, Ethical, and Biosafety Procedures

Stool samples were obtained from 110 children with diarrhea (54 boys and 56 girls) and 150 children without diarrhea (71 boys and 79 girls), aged from 1 month to 7 years old. Stool samples were collected from March 2008 through November 2010 at Hospital Municipal do Tatuapé, Hospital Infantil Cândido Fontoura, Hospital Infantil Menino Jesus and Centro Educacional Unificado (CEU-Butantã) in Sao Paulo city, SP, Brazil. Children did not display illnesses or comorbidities other than diarrhea, and they were not under antibiotic treatment for at least 3 months prior to sample collection. Diarrhea was defined as three or more unformed stools in the 24 h prior to enrollment. The Ethics Committee of the Biomedical Science Institute at the University of Sao Paulo approved this study (2006/743). All procedures followed the management of biosafety and biosecurity, since Biomedical Science Institute at São Paulo University guarantees excellent conditions for workers and population, and environmental protection from biohazards exposure.

### DNA Extraction

Total DNA from 200 mg of stool was obtained by using a QIAamp DNA Stool Mini Kit (QIAGEN) according to the manufacturer's instructions, resuspended in 100 μL of ultrapure water and stored at −80°C until use. DNA concentrations were determined by spectrophotometer (NanoDrop 2000, Thermo Scientific, USA), and 5 μL of each DNA sample were checked for integrity on 1% agarose gel.

### Identification of the Main Enteric Pathogens by qPCR

qPCR assays for genes *eae* (attaching and effacing lesions), *ipaH* (enteroinvasive mechanism), *lt* (heat-labile toxin*), st* (heat-stable toxin), *aggR* (aggregative fimbriae regulator), *invA* [inner membrane component of the *Salmonella* T3SS apparatus], and *ystA* (enterotoxin YstA *Yersinia* virulence plasmid) were carried out in duplicate and performed in a total volume of 25 μL, containing 2X TaqMan universal master mix (Applied Biosystems, USA), 10 μM of each primer, 10 μM of TaqMan probe, and 2 ng of DNA. Amplifications were performed in a thermal cycler programmed as follows: denaturation at 95°C for 10 min, followed by 45 cycles of two steps: denaturation at 95°C for 15 s and an annealing temperature at 60°C for 1 min. The primer/probe sets used are shown in Supplementary Table 1. A standard curve was also derived using 10-fold DNA dilutions from the reference strains with their respective primer pairs. Amplifications were adjusted to *R*^2^ > 0.900. A *P* < 0.05 was considered statistically significant. A sample was considered positive for a target gene when the detected fluorescence generated a curve above the background fluorescence, which was established by the Rotor-Gene Q6000 analytical software (Qiagen, Brazil).

### Differentiation of Enteric Pathogens by PCR

Samples positive for *eae* (*n* = 57) and *ipaH* (*n* = 15) by real-time PCR were further screened for the *eae, bfpA* (bundle-forming pilus for typical EPEC), *ipaH, lacY* (lactose permease), and *stx1* and *stx2* (Shiga toxins) genes by multiplex PCR according to the protocol of Aranda et al. (2004) and Pavlovic et al. (2011) for differentiation between EPEC/STEC and EIEC*/Shigella* samples.

### Characterization of the *eae*-positive Samples by High-Throughput Real-Time PCR

A LightCycler® 1536 (Roche, Meylan, France) was used to perform high-throughput real-time PCR amplifications as described previously (Delannoy et al., 2012b), except that 1 μL of sample DNA was used in each reaction for a final reaction volume of 2 μL. The thermal profile was modified as follows: 95°C for 1 min, followed by 45 cycles of 95°C for 0 s, and 58°C for 30 s. All ramp rates were set to 2°C/s. The primers and probes used targeted genes encoding intimin (*eae, eae*-alpha, *eae*-beta, *eae*-gamma, *eae*-epsilon, and *eae*-theta), O group-associated genes for the top 7 serogroups (*wzx*_O26_, *rfbE*__*O*157_, *wzy*__*O*145_, *wzx*__*O*103_, *wbdl*__*O*111_, *wzx*__*O*121_, *wzx*__*O*45_,), flagellar antigens H11, H19, H2, H28, H7, and H8 (*fliC*H11, *fliC*H19, *fliC*H2, *fliC*H28, *fliC*H7, and *fliC*H8), urease (*ureD*), effector proteins translocated by T3SS (EspL2 [*ent* {or *espL2*}], NleB [*nleB*], NleE [*nleE*], NleA [*nleA*], NleF [*nleF*], EfA1 [*efa1*], EspN [*espN*], EspK [*espK*], and EspM1 [*espM1*]), and other genetic markers including Z6065, *Z2098, Z2099, terE, hlyA, ehxA, and pagC* and markers related to the CRISPR loci *SP_O157_A, SP_O157_B, SP_O157_C, SP_O26_C, SP_O26_D, SP_O26_E* (Perelle et al., 2004; Fratamico et al., 2009, 2010; Bugarel et al., 2010; Delannoy et al., 2012a, 2013, 2015, 2016; Piazza et al., 2013). The *wecA* gene was used as a reference genetic marker for *E. coli* (Bugarel et al., 2011). An inhibition control (IC) was introduced in each sample to check for potential inhibition of the PCR reaction due to intrinsic characteristics of the sample. IC is a recombinant pBluescriptIISK+ plasmid containing the *dsb* gene from *Ehrlichia canis* (Michelet et al., 2014). The plasmid was added to each sample at a concentration of ~0.3 pg/μL. Primers and probe specific for the *Ehrlichia canis dsb* gene were used to detect IC (Michelet et al., 2014).

### Statistical Analyses

We compared the presence of pathogen between groups using the Fisher exact test. A comparison of bacterial loads in *eae*- and *aggR*-positive samples between samples from children with and without diarrhea was performed with a Mann-Whitney *U*-test. A *P* < 0.05 was considered statistically significant. All statistical analyses were performed in Stata v.12.1.

## Results

### Prevalence of Enteropathogens in Stool Samples Obtained From Children With and Without Diarrhea

A total of 260 stool samples (110 from children with diarrhea and 150 children without diarrhea) were collected from public hospitals and schools, respectively, in Sao Paulo city (the largest city in South America) from March 2008 through November 2010. The groups studied consisted of children between 1 month and 7 years old, representing much of childhood. Each group was further divided into 3 age groups: 0–11 months, 12–23 months, and older than 24 months. The pathogens detected through their representative genetic markers in the stool samples from children with and without diarrhea of different age are reported in Table 1.

**Table 1:** Distribution of virulence genes from enteric pathogens in fecal samples from children with and without diarrhea.

		**Asymptomatic group (*****n*** **=** **150)**				**Diarrhea group (*****n*** **=** **110)**				
**Pathogens detected**	**Genetic markers**	**0–11 months (*n* = 54)**	**12–23 months (*n* = 59)**	**> 24 months (*n* = 37)**	**Total**	**0–11 months (*n* = 55)**	**12–23 months (*n* = 50)**	**> 24 months (*n* = 5)**	**Total**	***P*-value**
STEC	eae, stx1/stx2	2	4	0	6	2	9	0	11	0.19
EAEC	aagR	15	9	1	25	10	8	0	18	0.016
EPEC Typ	eae, bfp	0	1	0	1	1	0	0	1	0.44
EPEC atyp	eae	0	0	0	0	3	1	0	4	0.033
ETEC	lt/st	3	1	0	4	1	3	0	4	0.38
EIEC	ipaH, LacY	0	1	2	3	4	3	1	8	0.088
STEC EAEC	eae, aggR, stx1/stx2	3	1	0	4	1	2	0	3	0.25
STEC EPECtyp	eae, bfp, stx1/stx2	1	1	0	2	3	5	0	8	0.049
STEC ETEC	eae, stx1/stx2, lt/st	1	0	0	1	0	0	0	0	0.13
EAEC ETEC	aggR, lt/st	3	3	0	6	1	0	0	1	0.013
EAEC EPECtyp	aggR, eae, bfp	2	1	0	3	0	0	0	0	0.02
EAEC EIEC	aggR, ipaH, LacY	0	0	0	0	1	0	0	1	0.182
EAEC EPECatyp	aggR, eae	0	0	0	0	1	1	0	2	0.098
EAEC Salmonella	aagR, invA	0	0	0	0	0	1	0	1	0.18
STEC EAEC ETEC	eae, stx1/stx2, aggR, lt/st	1	1	0	2	0	0	0	0	0.057
STEC EAEC EPECtyp	eae, aggR, stx1/stx2, bfp	0	1	0	1	2	0	1	3	0.2
STEC EPECtyp EIEC	eae, stx1/stx2, bfp, ipaH, LacY	0	0	0	0	1	0	0	1	0.18
STEC EPECtyp ETEC	eae, stx1/stx2, bfp, lt/st	0	0	0	0	1	0	0	1	0.18
STEC EPECtyp Yersinia	eae, stx1/stx2, bfp, invA	0	0	0	0	1	0	0	1	0.18
STEC ETEC EIEC	eae, stx1/stx2, lt/st, ipaH, LacY	0	0	0	0	1	0	0	1	0.18
STEC EPECtyp ETEC EIEC	eae, stx1/stx2, bfp, lt/st, ipaH, Lacy	0	0	0	0	0	2	0	2	0.098

*P < 0.05 was considered statistically significant*.

No genetic markers were detected in 39 of 110 (35%) children with diarrhea and 92 out of 150 (61%) without diarrhea. The presence of genetic markers associated with bacterial pathogens was significantly higher in stool samples from the diarrhea group (71/110) compared to the control (58/150) (*P* < 0.001). A significant increase in the number of samples positive for one pathogen was found in the diarrhea samples (46/110) compared to asymptomatic group (39/150) (*P* = 0.0036). Although we observed statistical differences between groups in samples with 3 or 4 pathogens, in both groups, the number of samples is low, not allowing further conclusions (Table 2).

**Table 2:** Association of enteric pathogens in fecal samples from children with and without diarrhea determined by virulence genes presence.

	**Asymptomatic group (*n* = 150)**	**Diarrhea group (*n* = 110)**	
**Number of pathogens detected**	**Total**	**Total**	***P*-value**
None	92	39	>0.001
Positive for 1 or more pathogens	58	71	>0.001
1 pathogen	39	46	0.0036
2 pathogens	16	16	0.1734
3 pathogens	3	7	0.0354
4 pathogens	0	2	0.0486

### Bacterial Loads in *eae*- and *aggR*-Positive Samples

**Figure 1:**
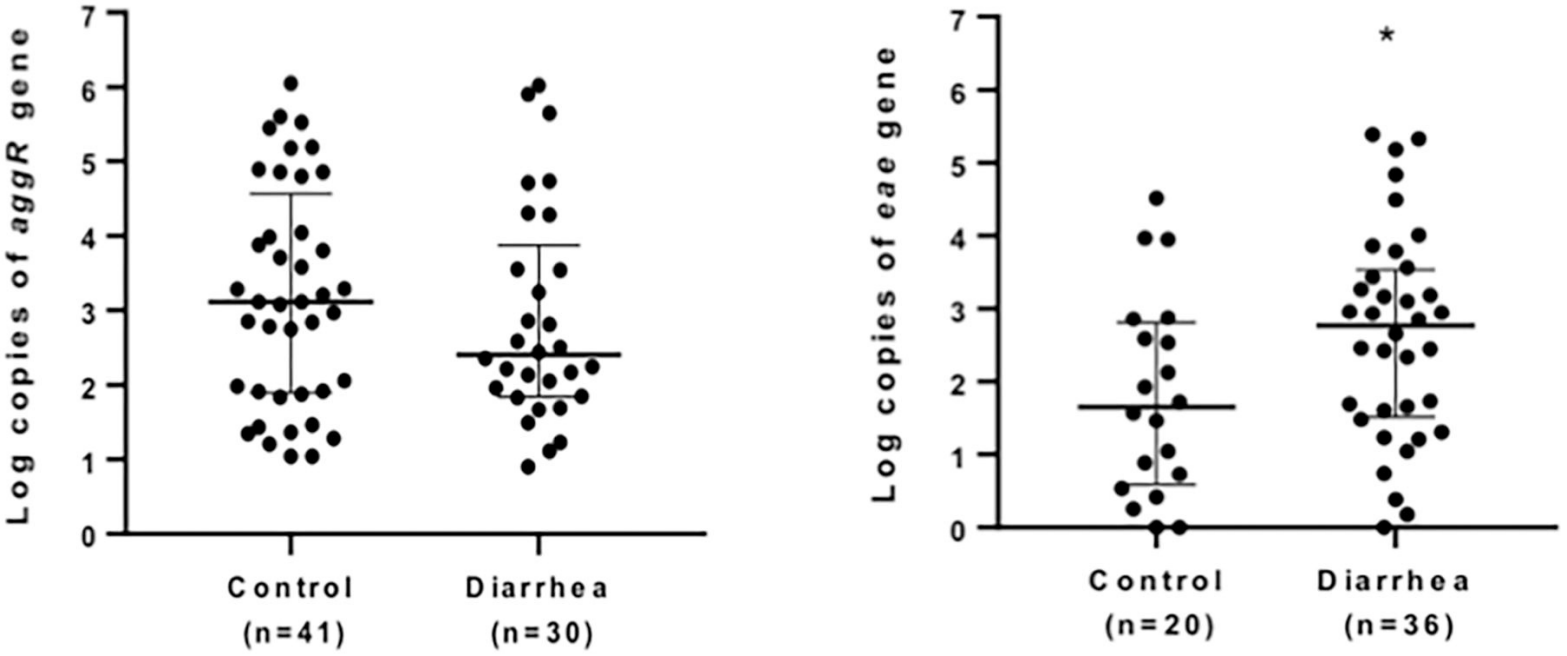
Quantification of *eae*- and *aggR*- markers in stool. Positive stool samples for *aggR* (left) or *eae* (right) markers from children with (diarrhea group) or without (control) diarrhea were analyzed by quantitative qPCR. Data are presented as median log copy number/mg of stool sample ± IQR. *P < 0.05 (Mann-Whitney *U*-test).

Quantification of bacterial loads was shown to be a promising tool to identify the etiological agent of diarrheal diseases. Of all the genes evaluated, *aggR* and *eae* were the markers most found in diarrheal and asymptomatic children (71 and 56 samples, respectively). We compared the copy number of *eae* and *aggR* genes in all the samples positive for these markers in both diarrhea and asymptomatic groups (Figure 1). A significantly higher copy number of *eae* was found in diarrhea samples (*P* < 0.039); no differences in *aggR* copy number were found between groups (*P* = 0.43).

### Prevalence of EHEC-Associated Virulence Markers in *eae*-positive Stool Samples From Children With and Without Diarrhea

In the control group, we detected 13% *eae-*positive samples and 11% *stx*-positive samples, while in the diarrhea group, 34% *eae-*positive samples, and 27%-*stx* positive samples were present, and thus, the presence of these genes were significantly higher in the diarrhea group. Surprisingly, our results indicated higher percentages of the majority of the additional genetic markers that are reported to be mostly predominant in EHEC in asymptomatic children (Table 3). Comparing all genetic markers, no differences were observed between the groups (*P* = 0.312), but the presence of the genes *stx2, ehxA, nleB, nleE, nleF, espM1, pagC, ent, efa1*, espV, Z2098, Z2099, Z6065, *terE, ureD*, and SP_O157 A, B, and C were statistically significant in the asymptomatic children compared to the diarrhea groups (*P*-values varying from 0.0001 to 0.0035) (Table 3).

**Table 3:** Virulence profile determination of *eae*+ *Escherichia coli* from fecal samples of children with and without diarrhea^*^.

**Genetic markers**	**32 fecal samples with diarrhea (%)**	**19 fecal samples without diarrhea (%)**	***P*-value**
*bfp*	1 (3.1)	0 (0)	0.2192
*stx1*	1 (3.1)	3 (15.8)	0.0513
*stx2*	8 (25.0)	12 (63.2)	0.0035
*ehxA*	6 (18.8)	13 (68.4)	0.0002
*hlyA*	12 (37.5)	7 (37.0)	0.4858
*nleA*	5 (15.6)	2 (10.5)	0.3043
*nleB*	12 (37.5)	14 (73.7)	0.0062
*nleE*	12 (37.5)	15 (79.0)	0.0020
*nleF*	13 (40.6)	15 (79.0)	0,0039
*espM1*	12 (37.5)	14 (73.7)	0.0062
*pagC*	6 (18.8)	15 (79.0)	0.0001
*ent*	7 (22.0)	15 (79.0)	0.0001
*efa1*	3 (9.4)	9 (47.4)	0.0010
*espK*	6 (18.8)	4 (21.0)	0.4242
*espN*	1 (3.1)	1 (5.3)	0.3477
*espV*	8 (25.0)	13 (68.4)	0.0012
Z2098	7 (22.0)	12 (63.2)	0.0016
Z2099	12 (37.5)	16 (84.2)	0,0006
Z6065	12 (37.5)	13 (68.4)	0.0164
*terE*	29 (91.0)	18 (94.7)	0.3151
*ureD*	14 (44.0)	15 (79.0)	0.0073
SP_O157-A	6 (18.8)	11 (58.0)	0.0021
SP_O157-B	1 (3.1)	7 (37.0)	0.0007
SP_O157-C	6 (18.8)	11 (58.0)	0.0021
O26_C	4 (12.5)	1 (5.3)	0.2017
O26_D	2 (6.2)	1 (5.3)	0.4474

*P < 0.05 was considered statistically significant*.

* *General P value between groups 0.312*.

## Discussion

A better knowledge of the distribution of pathogens linked to stool contamination is important for evaluating the pathogen loads in the environment as well as the associated health risks (Zhang et al., 2016). Over the last few decades, new molecular methods have become essential in allowing the rapid detection and characterization of pathogens (Miliwebsky et al., 2016).

The present study was performed to determine the molecular epidemiology of enteropathogens among children under seven years old with and without acute gastroenteritis in Sao Paulo, SP, Brazil. Virulence markers for DEC pathotypes were largely detected confirming some other surveys in Latin America and China (Ochoa et al., 2009; Moreno et al., 2010; Zhang et al., 2016; Lima A. A. M et al., 2019; Lima F. M. et al., 2019). Different results were reported in Western Kenya among bacterial pathogens, where two species of *Shigella* were the most prevalent (Swierczewski et al., 2013).

Our results showed that the genes encoding virulence factors for *Salmonella* and *Yersinia* were detected in very low frequencies, in agreement with another survey in counties near Sao Paulo city (Lima F. M. et al., 2019).

Herein, DEC-associated genetic markers were detected in 71 out of 110 (65%) children with diarrhea and 58 out of 150 (39%) without diarrhea and significant differences were observed between the two groups. These results are in agreement with other studies in South American countries. In Northeast Brazil, the presence of EPEC and EAEC were consistently associated with diarrhea (Maranhão et al., 2008; Moreno et al., 2010). Furthermore, EAEC and STEC showed by multivariate logistic regression analysis significant odds of being associated with a risk for diarrheal diseases as well as norovirus, adenovirus, rotavirus, and *Giardia* infection. Furthermore, EAEC enteric infections are associated with a high clinical severity score plus moderate-to-severe dehydration (Lima A. A. M et al., 2019).

The shifting in DEC epidemiology has been described in different studies and geographical regions (Pérez et al., 2010; Torres, 2017; Imdad et al., 2018; Pérez-Corrales and Leandro-Sandí, 2019) including Brazil, where until the 1990s, EPEC, mainly the typical EPEC serotypes producing EPEC adherence factor (EAF), was the main cause of infantile diarrhea (Gomes et al., 1991; Rosa et al., 1998; Regua-Mangia et al., 2004), and also *Salmonella* species (8%), ETEC (7%), and *Shigella* species (5%) were associated with diarrhea (Gomes et al., 1991), but it seems that they are becoming more and more rare, as presented herein and described elsewhere (Franzolin et al., 2005; Bueris et al., 2007; Moreno et al., 2010; Lima A. A. M et al., 2019; Lima F. M. et al., 2019; Ori et al., 2018).

The distribution of genes encoding either for one single pathogen in the diarrhea group (66%) and in control (67%) or in multiple coinfections, 34 and 33%, respectively, was similar in the present study. However, except for *Salmonella* plus *E. coli* and *Yersinia* plus *E. coli*, we cannot assure that we detected different strains, since they were not isolated from stools. Nevertheless, coinfections have been described, suggesting associations among potential enteropathogens in the etiology of diarrhea (Orlandi et al., 2006; Lima F. M. et al., 2019). Indeed, some hybrid strains possessing typical genes from different pathotypes (heteropathogens) have recently emerged (Santos et al., 2020). For example, the O104:H4 strain associated with the severe German outbreak in 2011 was a hybrid of both EAEC and EHEC (Brzuszkiewicz et al., 2011). More recently, enterohemorrhagic *E. coli* hybrid pathotype O80:H2 has emerged in Europe as an important serotype responsible for hemolytic uremic syndrome (HUS) associated with extraintestinal infections in children (Cointe et al., 2018). Also, coinfections have been reported, detecting in stool samples the presence of EPEC and EAEC and *S. enterica houtenae* and EAEC (Lima F. M. et al., 2019).

The incidence of intestinal infection caused by STEC is low in the Brazilian population (de Souza et al., 2011; Ori et al., 2018; Lima F. M. et al., 2019). Thus, it was surprising to find more EHEC-associated genetic markers in the asymptomatic group than in the diarrhea group, since these genetic markers are usually more associated with *E. coli* strains of greater clinical significance (Delannoy et al., 2012a,b, 2016; Piazza et al., 2013). This suggests that these potential EHEC strains are not the etiological agent of diarrhea in this sampled group, which remains to be investigated.

Current methods for either screening or detecting of enteropathogens by PCR, multiplex PCR, and qPCR have been used worldwide, given the high sensitivity and specificity, which are satisfactory parameters to ensure an adequate diagnosis (Panchalingam et al., 2012). However, the presence of several pathogens in diarrhea samples, as we observed, and the high frequency of enteropathogens in asymptomatic children has made it difficult to use these methods as a tool to identify the etiological agent of diarrhea.

Here, we found a significantly higher copy number of *eae* in diarrhea samples compared to samples from asymptomatic children, suggesting that LEE-containing pathogens could be the etiological agents responsible for diarrhea in children. Even though we did not find differences between groups in the number of copies for *aggR*, our data must be complemented with a larger number of samples and/or selection of other EAEC markers to support the use of qPCR for molecular diagnosis for this pathogen.

Our study had the following limitations. The number of samples was limited and we did not search for other relevant pathogens, such as rotavirus, norovirus, protozoa, and helminthes. For bacterial loads, we only evaluated two markers, which might be insufficient, considering that DEC harbors several virulence determinants that can be used as a target for diagnosis. Another limitation of the study was that we did not isolate the pathogenic strains. This would be helpful to identify if asymptomatic children might shed the bacteria and possibly be contagious.

In conclusion, we found that the presence of genetic markers associated with bacterial pathogens was significantly higher in stool samples from the diarrhea group compared to the control. Also, compared to samples from asymptomatic children, there was a significantly higher copy number of the *eae* gene in diarrhea samples. The data presented herein demonstrated that asymptomatic children of an urban area in Brazil might be enteropathogen reservoirs, especially for STEC, thus favoring their widespread.

## Data Availability Statement

All datasets generated for this study are included in the article/Supplementary Material.

## Ethics Statement

The studies involving human participants were reviewed and approved by Ethics Committee of the Biomedical Science Institute at the University of Sao Paulo. Written informed consent to participate in this study was provided by the participants' legal guardian/next of kin.

## Author Contributions

VM, VN, SD, PF, RP, and MA-C participated in the design of the study. VM, VN, SD, PF, MF, RP, and MA-C participated in data analysis. MF, RP, SD, and PF participated in writing the manuscript. VM and VN carried out the qPCR experiments. GA carried out the multiplex PCR experiments. SD and PF carried out the high-throughput real-time PCR experiments. All authors read and approved the final manuscript.

## Conflict of Interest

The authors declare that the research was conducted in the absence of any commercial or financial relationships that could be construed as a potential conflict of interest.
