# Supplementary material for: Prevalence of Enteropathogens and Virulence Traits in Brazilian Children With and Without Diarrhea
Source: Front Cell Infect Microbiol. 2020 Sep 25;10:549919. doi: 10.3389/fcimb.2020.549919 (PMC7545120; doi:10.3389/fcimb.2020.549919)
Supplement: Supplementary file 1 [file Table_1.doc]

Table S1. Nucleotides sequences and conditions used to quantify bacterial groups or species by real time PCR.

| Microorganisms  (genes) | Sequences  5′ 3′ | Tm (°C) | Strains^§^ | References |
| --- | --- | --- | --- | --- |
| *Escherichia coli* LEE-containing pathogens | F: TGT TGC TTT GTT TAA TTC YGA TAA GC  R: GGA ATC GGA GTA TAG TTT ACA CCA A | 60 | *E. coli*  E2348/69 | Iijima et al. (2007) |
| *(eae)* | P: r-AGT CGA ATC CTG GTG CGG C-q |  |  |  |
|  |  |  |  |  |
| *Escherichia coli* ETEC | F: AAG AGC GGC GCA ACA TTT  R: CAA TGG CTT TTT TTT GGG AGT CT | 60 | *E. coli*  H10407 | Iijima et al. (2007) |
| (*lt*) | P: r-AGG TCG AAG TCC CGG GCA GTC AA-q |  |  |  |
|  |  |  |  |  |
| *Escherichia coli* ETEC | F: AGA ATC AGA ACA AAT ATA AAG GGA ACT GT | 60 | *E. coli* | Iijima et al. (2007)) |
|  | R: CCT GAA AGC ATG AAT AGT AGC AAT TACT |  | H10407 |  |
| (*st*) | P: r-AGC ACC CGG TAC AAG CAG GAT TAC AAC A-q |  |  |  |
|  |  |  |  |  |
| *Escherichia coli* EAEC | F: ATG CCC TGA TGA TAA TAT ACG GAA TAT  R: TCA GCA TCA GCT ACA ATT ATT CCT TT | 60 | *E. coli*  042 | Iijima et al. (2007) |
| (*aggR)* | P: r-AAA AGT AGA TGC TTG CAG TTG TCC GAA TTG G-q |  |  |  |
|  |  |  |  |  |
| *Escherichia coli* EIEC/ *Shigella* spp. | F: CCT TTT CCG CGT TCC TTG  R: CGG AAT CCG GAG GTA TTG C | 60 | *Shigella sonnei*  (2) 15.05.07 | Thiem et al., (2004) |
| (*ipaH*) | P: r-CGC CTT TCC GAT ACC GTC TCT GCA-q |  |  |  |
|  |  |  |  |  |
| *Salmonella* spp. | F: GCG TTC TGA ACC TTT GGT AAT AA  R: CGT TCG GGC AAT TCG TTA | 60 | *Salmonella* spp.  (2) 2009 | Daum et al. (2002) |
| (*invA*) | P: r-TGG CGG TGG GTT TTG TTG TCT TCT-q |  |  |  |
|  |  |  |  |  |
| *Yersinia enterocolitica* | F: AAT GCT GTC TTC ATT TGG AGC  R: ATC CCA ATC ACT ACT GAC TTC | 60 | *Yersinia enterocolitica* | Zheng et al. (2007) |
| (*ystA*) | P: r-CAA GCA AGC TTG TGA TCC TCC G-q |  | O:3 |  |
|  |  |  |  |  |

§: Strains used for the standard curve construction.

F = forward primer; R = reverse primer; P = probe; r = reporter (FAM); q = quencher (TAMRA).

**References table S1**

Daum LT, Barnes WJ, McAvin JC, Neidert MS, Cooper LA, Huff WB, Gaul L, Riggins WS, Morris S, Salmen A, Lohman KL. Real time PCR detection of *Salmonella* in suspect foods from a gastroenteritis outbreak in Kerr County, Texas. J. Clin. Microbiol. 40: 3050-3052, 2002.

Iijma Y, Tanaka S, Miki K, Kanamori S, Toyokawa M, Asari S. Evaluation of colony-based examinations of diarrheagenic *Escherichia coli* in stool specimens: low probability of detection because of low concentrations, particularly during the early stage of gastroenteritis. Diag. Microbiol. Infect. Dis. 58: 303-308, 2007.

Thiem VD, Sethaburt O, Seidlein L, Tung TV, Canh DG, Chien BT, Tho LH, Lee H, Houng HS, Hale TL, Clemens JD, Mason C, Trach DD. Detection of *Shigella* by a PCR assay targeting the *ipaH* gene suggests increased prevalence of Shigellosis in Nha Trang, Vietnam. J. Clin. Microbiol. 42: 2031-2035, 2004.

Zheng H, Wang J, Sun Y, Jiang B. Clinical isolation and characterization of *Yersinia enterocolitica* in China using Real Time PCR and culture method. Digestion. 75: 199-204, 2007.
